# Evaluation of Type I endoleak after endovascular aneurysm repair for abdominal aortic aneurysm using component quantification of energy loss: a preliminary feasibility study

**DOI:** 10.3389/fcvm.2026.1849668

**Published:** 2026-08-21

**Authors:** Ran Tao, Qiming Liu, Weihao Guo, Lingshang Kong, Wenbin Wang, Xudong Jiang, Peng Wu, Yeben Qian

**Affiliations:** 1 General Surgery Department, The First Affiliated Hospital of Anhui Medical University, Hefei, China; 2 General Surgery Department, The Second Affiliated Hospital of Anhui Medical University, Hefei, China; 3 Precision and Intelligence Medical Imaging Lab, Beijing Friendship Hospital, Capital Medical University, Beijing. China

**Keywords:** abdominal aortic aneurysms, computational fluid dynamics, endovascular aneurysm repair, energy loss, type i endoleak

## Abstract

**Objective:**

This study aimed to perform hemodynamic simulations using patient-specific models of abdominal aortic aneurysms (AAA) following endovascular aneurysm repair (EVAR) to investigate the association between energy loss (EL) and Type I endoleak.

**Methods:**

Pre- and post-EVAR models were reconstructed from computed tomography angiography (CTA) for four patients with Type I endoleak and four without. Component quantification of EL, along with wall shear stress (WSS)-related hemodynamic parameters, were employed to evaluate the potential of endoleak.

**Results:**

Endoleaks following EVAR tended to occur at the junction of stent grafts, where high time-averaged wall shear stress (TAWSS), high turbulence EL, and high mean flow EL were observed. The turbulence EL values for the main stents, left iliac branch stents, and right iliac branch stents in the endoleak group were 516 ± 183*10^−6^ W, 828 ± 195*10^−6^ W, and 609 ± 193*10^−6^ W, respectively, and the mean flow EL for those regions were 363 ± 125*10^−6^ W, 631 ± 138*10^−6^ W, and 352 ± 162*10^−6^ W, respectively. Compared with the no endoleak group, the endoleak group exhibited significantly higher turbulence EL and mean flow EL at the iliac branch and main stent, whereas no significant difference in TAWSS was found.

**Conclusion:**

Component quantification of EL demonstrated preliminary feasibility for evaluating Type I endoleak in patients with EVAR for AAA, demonstrating greater advantages over WSS-related hemodynamic parameters. This method may provide valuable support for clinicians in assessing surgical prognosis.

## 1 Introduction

Endovascular aneurysm repair (EVAR), recognized as the most established surgical therapy for abdominal aortic aneurysms (AAA), offers significant advantages including minimal trauma and high early survival rates, and is now widely adopted in clinical practice (1–4). Nevertheless, postoperative endoleak remains a major complication that may lead to persistent aneurysm expansion and significantly compromise long-term outcomes (5, 6). In recent years, clinicians have conducted extensive research on the risk factors associated with endoleak (7–9). There is a general consensus that stent-related characteristics and complex aneurysm anatomy constitute primary contributors to endoleak development (10, 11). Based on the source of blood flow, endoleaks are primarily classified into Types I through V, as shown in Table 1 (12).

**Table 1:** Classification of endoleaks.

Type of endoleak	Definition
Type I endoleak	Failure to create an adequate circumferential seal
	Ia—proximal attachment site
	Ib—distal attachment site
	Ic—common iliac artery
Type II endoleak	Backflow of blood from aortic collaterals into the aneurysmal sac
Type III endoleak	Endoleak secondary to structural failure of endograft
	IIIa—component disconnection
	IIIb—stent fabric disturbance
Type IV endoleak	Related to graft fabric porosity
Type V endoleak (endotension)	Continued high intrasac pressure following EVAR without evidence of continued aneurysm sac perfusion

With growing insights into the pathomechanism underlying endoleaks, hemodynamic factors have emerged as a key focus of current research (13, 14). It is widely recognized that persistent hemodynamic forces within the aneurysm sac serve as a critical initiating factor for both endoleak occurrence and potential rupture (15, 16). From a hemodynamic perspective, endoleaks can be divided into direct endoleaks and indirect endoleaks. Direct endoleaks are defined as the direct transmission of arterial pressure to the aneurysm wall, a condition associated with a high risk of rupture, among which Type I is the most prevalent (12). Over the past two decades, computational fluid dynamics (CFD) has become an indispensable tool in evaluating AAA rupture risk and predicting outcomes following EVAR (17–21). Li et al. (19) characterized the pressure shielding capacity of stent-grafts post-EVAR, demonstrating its spatiotemporal influence on endoleak development. Lu et al. (20) identified a significant association between elevated local wall shear stress (WSS) and endoleak susceptibility, proposing that localized reinforcement of stent-graft mechanical strength may mitigate risk in vulnerable regions. Domanin et al. (21) integrated longitudinal stent displacement measurements with time-averaged wall shear stress (TAWSS) analyses across different stent materials, revealing that nitinol-based stents exhibit superior stability, which were associated with a lower incidence of endoleak. While high WSS and intraluminal pressure have consistently been implicated as key contributors to endoleak pathogenesis through analysis of mural hemodynamic parameters, broader alterations in global flow field dynamics that may drive or modulate these localized phenomena were overlooked.

Hemodynamic patterns within AAAs are highly complex, frequently characterized by vortices, flow reversal, and secondary flows (22–25). In a retrospective study of 106 AAA patients, intraluminal flow was categorized into three types, and the type with vortex-dominant flow structure was shown to be strongly associated with increased rupture risk (24). Energy loss (EL), as a quantitative metric reflecting the evolution of vortex, has increasingly drawn attention in hemodynamic research (26–30). The total energy dissipation rate can be statistically divided into two parts: mean flow EL and turbulence EL. Mean flow EL corresponds to hydraulic losses arising from time-averaged velocity gradients, predominantly occurring at the vessel wall and attributable to frictional interactions between blood and the endothelial surface. In the aorta, where flow is inherently turbulent, EL manifests across both spatial and temporal domains. Turbulence EL quantifies the rate of viscous dissipation per unit mass and time during pulsatile flow deformation, primarily localized in regions of flow disturbance. Consequently, turbulence EL serves as a key indicator for characterizing the overall hemodynamic instability of aortic flow. Qiao et al. (29) demonstrated that energy loss mainly located to regions with pathological structural alterations, such as aortic stenosis or aneurysmal dilation. In our prior study, we further established significant associations between both turbulence energy loss and mean flow energy loss and the risk of aneurysm rupture (30). Given that post-EVAR endoleak shares hemodynamic and biomechanical characteristics with aneurysm rupture, investigating intrastent energy loss following EVAR holds substantial potential for elucidating the underlying hemodynamic mechanisms of endoleak.

In this study, 8 patients with AAA who underwent EVAR at the Second Affiliated Hospital of Anhui Medical University were retrospectively enrolled. Based on postoperative computed tomography angiography (CTA), patients were separated into two groups within or without Type I endoleak. Using patient-specific CFD simulation, EL in both the aneurysm sac and the endograft lumen was quantified pre-EVAR and post-EVAR. By integrating these metrics with conventional hemodynamic parameters, including flow patterns, TAWSS and oscillatory shear index (OSI), the association between EL and endoleak risk was revealed, thereby advancing the development of a more comprehensive prediction for post-EVAR endoleak.

## 2 Material and methods

### 2.1 Models

This study was conducted in accordance with the provisions of the Declaration of Helsinki, which was approved by the Ethics Review Committee of the Second Affiliated Hospital of Anhui Medical University. Postoperative EVAR patients from October 2020 to September 2025 were retrospectively screened. Inclusion criteria: (1) infrarenal AAA treated with EVAR; (2) postoperative CTA with adequate image quality; (3) complete clinical records. Exclusion criteria: (1) severe metal artifacts or incomplete coverage; (2) prior reintervention before index CTA; (3) non-Type I endoleak only; (4) poor segmentation feasibility. 8 patients in this study were divided into Group A (endoleak) and Group B (no endoleak), and the patient subject data was shown in Table 2.

**Table 2:** Patient specifics.

Group	Case	Gender	Age (y)	Aneurysm maximum diameter (mm)	Aneurysm volume (cm^3^)	Aneurym topology	Aneurysm neck angle	Iliac artery stent-grafts type
Group A (endoleak)	CaseA1	M	81	52.8	446.81	Fusiform	130	Bifurcated Stent-graft
	CaseA2	M	86	48.5	79.513	Fusiform	131	Crossed-limb
	CaseA3	W	67	54.4	104.76	Saccular	156	Crossed-limb
	CaseA4	W	69	51.5	110.75	Saccular	137	Bifurcated Stent-graft
Group B (no endoleak)	CaseB1	M	74	42.6	73.224	Saccular	125	Bifurcated Stent-graft
	CaseB2	M	55	30.4	30.357	Saccular	153	Bifurcated Stent-graft
	CaseB3	M	61	83.0	165.73	Fusiform	137	Bifurcated Stent-graft
	CaseB4	M	71	67.7	150.28	Saccular	163	Crossed-limb

The detailed information concerning endoleaks in Group A was presented in Table 3. The patient-specific preoperative and postoperative models were reconstructed based on CTA data in the commercial software Mimics 20.0 (Materialise, Plymouth, Michigan). Surface processing was performed in Geomagic 20.0 (3D Systems, Inc, Rock Hill, SC, USA) using constrained smoothing to reduce excessive self-intersections, spikes, and highly creased edges while preserving key anatomical areas (proximal/distal sealing zones, graft junctions, and branch ostia), which was believed to bolster the robustness of simulations and mitigate associated errors. Pre/post smoothing consistency was checked by global and local geometric descriptors (e.g., lumen volume, surface area, and landmark diameters). The error was controlled to be within 1%. Models exceeding preset tolerance thresholds were reprocessed.

**Table 3:** Details of endoleaks.

Case	Detection time (post-EVAR）	Whether sac enlargement	Clinical management	Whether reintervention
CaseA1	2 months	+	Surgical treatment	+
CaseA2	6 months	−	Conservative follow-up	−
CaseA3	3 months	+	Surgical treatment	+
CaseA4	3 months	+	Conservative follow-up	−

**Figure 1:**
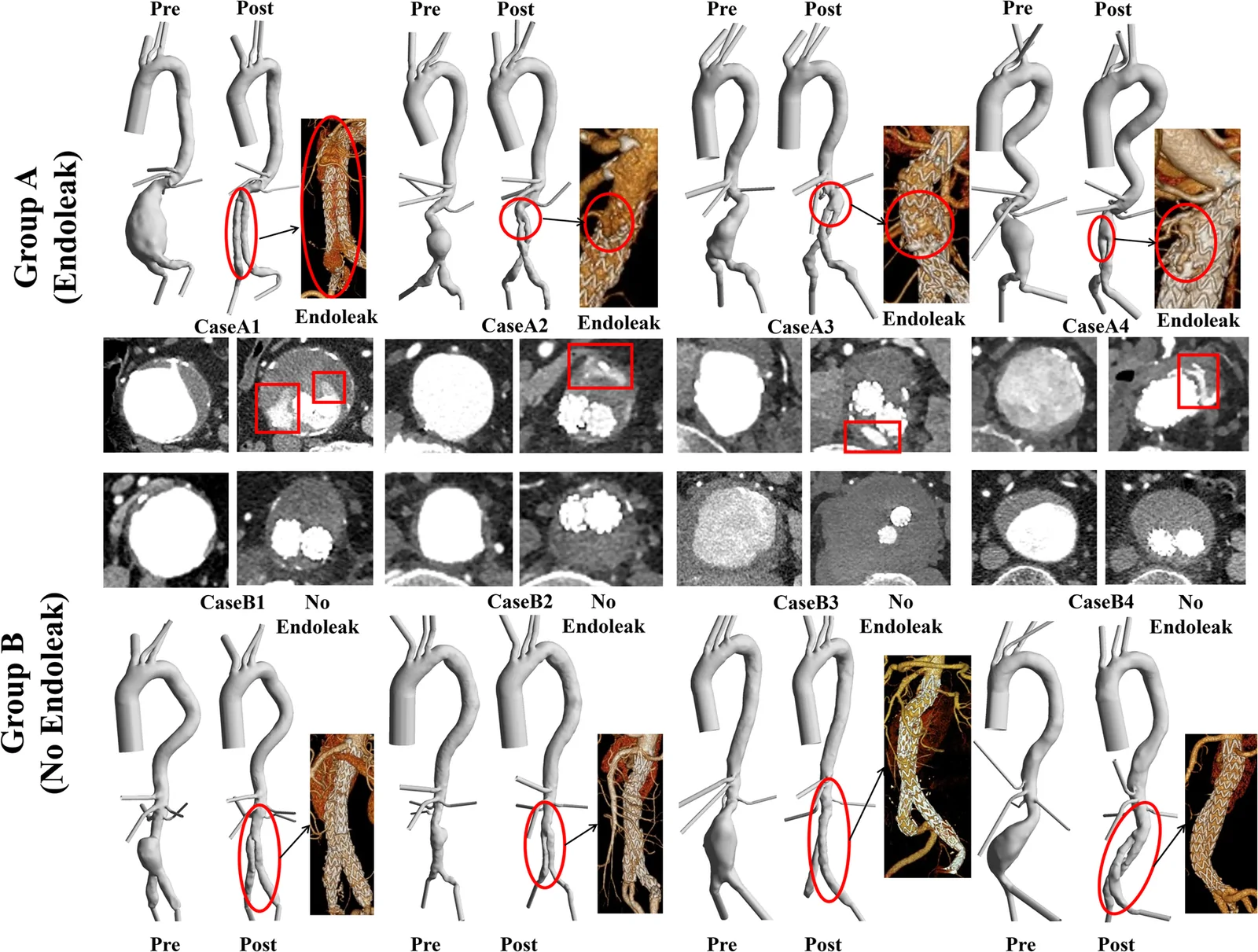
Patient-specific models: in Group A, endoleak was identified at both the proximal and distal ends of the right iliac branch stent-graft in CaseA1. In CaseA2, the endoleak was located at the proximal end of the main stent. And in both CaseA3 and CaseA4, the endoleak was located at the junction between the iliac branch and the main body stent-grafts. Notably, these endoleaks were marked but not reconstructed and the corresponding CTA cross-sectional images are provided to corroborate the anatomical localization. In Group B, the entire stent-grafts were fully segmented and visualized, with no evidence of endoleak. Furthermore, all patient-specific models underwent surface smoothing to increase the robustness during simulations.

Endoleak arises when blood extravasates from the interior of the stent or vessel to the exterior. Subjected to pulsatile forces, its topological structure undergoes dynamic changes. Since CTA only captures the morphology of endoleak at a single time point, reconstructing the model based on such static data could introduce unforeseen biases or confounding effects on the study results. Therefore, this study exclusively simulated models representing the pre-endoleak state and cross-referenced the simulation outcomes with the clinically documented locations of endoleak occurrence, so the endoleak was marked but intentionally excluded from the postoperative models to isolate the impact on hemodynamic performance. The locations of the endoleak and the key structural features of the patient-specific models were illustrated in Figure 1.

### 2.2 Mesh & boundary conditions

**Figure 2:**
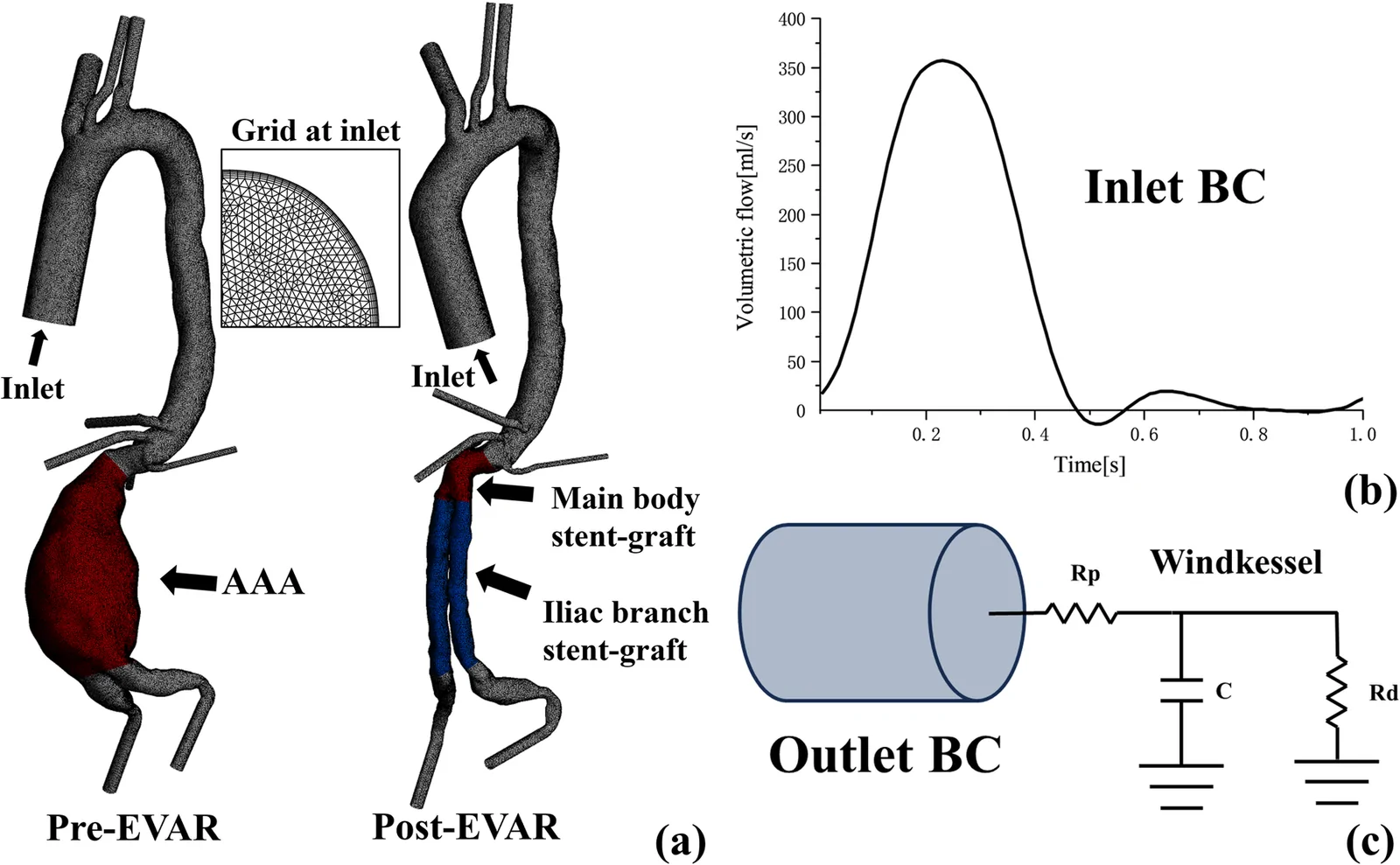
Mesh and boundary conditions: **(a)** computational domain and mesh of CaseA1, with local grid at the aortic inlet showing five boundary layers. AAA, main body stent-graft and iliac branch stent-grafts were segmented separately in blue and red parts; **(b)** The inlet velocity profile; **(c)** The Windkessel RCR outlet boundary conditions.

This study aims to investigate energy loss within the stent-grafts following EVAR and its potential association with Type I endoleak. During EVAR, the stent-grafts consist of the main body and the iliac artery branch. To enable detailed hemodynamic assessment, the aneurysm sac, main body stents, and iliac branch stents were segmented separately, allowing for comparative analysis of preoperative and postoperative hemodynamic parameters. Three grids of 9.38 million, 5.25 million, and 2.97 million were generated for the pre-EVAR model of CaseA1 for a mesh sensitivity analysis. The grid of 5.25 million was confirmed as the proper mesh for the simulation (results are in the “Results” section). Grids of 3.12–8.62 million were generated for other models with similar setup as the grid of 5.25 million by using commercial software Ansys Meshing (Ansys, Inc, Canonsburg, PA, USA). 5 grid layers were added to all the arterial walls to properly resolve the boundary layer to guarantee accuracy in hemodynamic parameter prediction, such as TAWSS and component quantification of energy loss, as shown in Figure 2. A standardized time-varying flow waveform was applied at the inlet (31), while Windkessel RCR boundary conditions were implemented at the outlet to physiologically represent downstream vascular impedance. The converged 3 EWM parameters were obtained through 5–6 rounds of patient-specific iterative calculation following our previous study (32, 33). All the walls were assumed to be rigid with no slip conditions.

### 2.3 Numerical simulation

In this study, all the simulations were transient and conducted using the commercial software Ansys Fluent (Ansys, Inc, Canonsburg, PA, USA). Blood was regarded as incompressible Newtonian fluids, with density of 1,055 kg/m^3^, and dynamic viscosity of 3.5 × 10^−3^ Pa.s. Although blood flow in large portions of the abdominal aorta is generally laminar or transitional, complex local flow features, including flow separation, recirculation, secondary flow, and vortex formation, may occur near stent-graft junctions, the iliac bifurcation, and regions with abrupt geometric curvature after EVAR. These disturbed flow structures are relevant to the estimation of mean flow EL and turbulence EL. The RNG k-*ε* model provides engineering robustness for evaluating complex recirculating flows and vortex dissipation, and was therefore adopted in the present hemodynamic simulations. A second-order implicit backward Euler scheme was chosen for temporal discretization, with a fixed time-step of 10 ms so that per cardiac cycle was resolved using 100 time-steps. Maximum 50 sub-iterations were used for each physical time step, and the maximum RMS residual was set to 10^−5^ as a convergence criterion. Based on the statistically converged flow field of 10 cardiac cycles, another 10 cardiac cycles of time-averaged simulation were conducted to obtain the conventional hemodynamic parameters related to TAWSS. Energy loss was solved by compiling a user-defined function (UDF) in Fluent, which was the same as in our previous study (30). The total energy dissipation rate $\left(\varepsilon_{tot}\right)$ can be statistically divided into two parts: mean flow EL $\left(\varepsilon_{mean}\right)$ and turbulence EL $\left(\varepsilon_{turb}\right)$, which is defined as:

$$\varepsilon_{tot}=\varepsilon_{mean}+\left\langle \varepsilon_{turb}\right\rangle =2\nu \left\langle S_{ij}\right\rangle \left\langle S_{ij}\right\rangle +2\nu \left\langle S_{ij}^{\prime }S_{ij}^{\prime }\right\rangle .$$

All computations were carried out on a 192-core cluster equipped with 16 Intel Xeon E5-2680 v3 CPUs. The simulations normally converged within 10 h.

### 2.4 Statistical analysis

Given the small sample size, statistical analysis was predefined as exploratory. Group comparisons were performed with small-sample-appropriate methods and interpreted conservatively. Group differences were assessed using non-parametric tests (exact Mann–Whitney U), and effect sizes with 95% confidence intervals were reported. *P*-values were interpreted cautiously and not used as sole evidence. We prioritized effect magnitude and uncertainty (with confidence intervals where applicable) rather than relying exclusively on *p*-values. Reported significance values are provided for completeness, but no confirmatory inference is claimed. All analyses were conducted using commercial software SPSS 31.0, two-sided tests.

## 3 Results

### 3.1 Grid sensitivity analysis

The grid sensitivity analysis was conducted for the pre-EVAR model of CaseA1. The mean flow EL at aneurysm were compared to the results predicted with the fine mesh. The mean flow Els were shown in Table 4. The difference between the results of the “Middle” and “Fine” mesh was almost negligible compared with the “Coarse” mesh. Therefore, the middle grid will be employed for the studies, and similar set up was employed when generating grids for other cases.

**Table 4:** Results of grid sensitivity analysis.

Mesh	Cells (×10^6^)	Mean flow EL (10^−6*^ W)	Error of mean flow EL (%)
Coarse	2.97	553	5.9
Middle	5.25	530	1.5
Fine	9.38	522	/

Error of Mean flow EL (%), defined as | Mean flow EL—Mean flow EL_0_|/ Mean flow EL_0_, where Mean flow EL_0_ is the the mean flow EL at aneurysm predicted with the fine mesh.

### 3.2 Flow patterns

**Figure 3:**
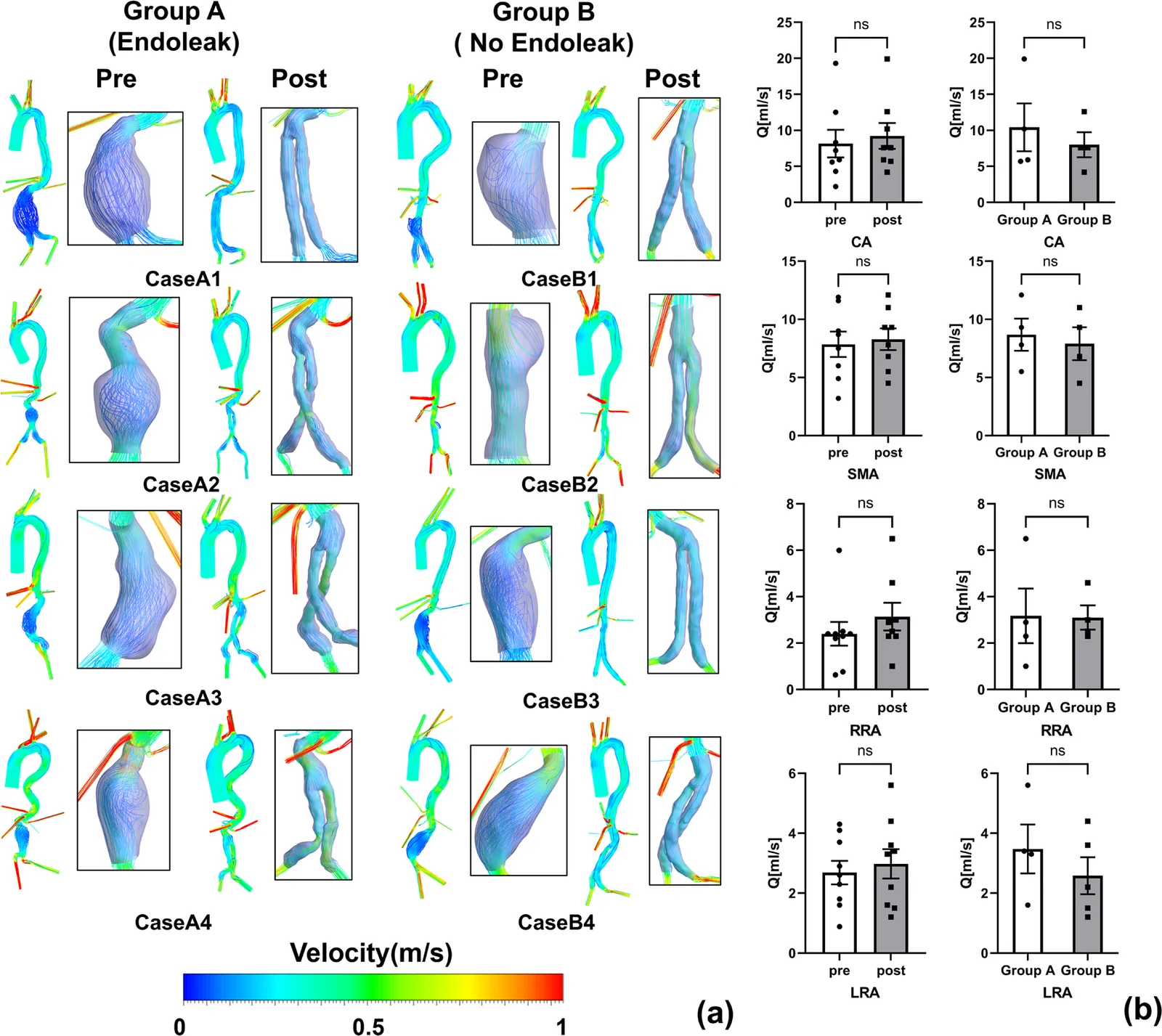
Flow patterns **(a)** Streamlines: the stent junction regions in the endoleak group generally demonstrated increased flow velocity and disturbance; **(b)** the velocity of visceral branch flow, with no significant difference in post-EVAR models between two groups. [Data was shown as mean ± standard deviation (SD)].

The flow pattern within the AAAs was chaotic and disorganized, characterized by extensive backflow and vortices, with the velocity significantly slower than that in other segments. Following EVAR, the blood flow fields within the main stents and iliac branch stents became stable, predominantly exhibiting laminar flow patterns, as illustrated in Figure 3. Notably, compared to the non-endoleak group, the stent junction regions in the endoleak group generally demonstrated increased flow velocity and disturbance. Analysis of visceral branch flow velocity pre- and post-EVAR revealed no statistically significant changes in velocity across the perioperative period. Additionally, no significant difference in post-EVAR flow velocity was observed between the endoleak and non-endoleak groups. These findings suggested that the velocity of branch arteries maybe not a contributing factor to endoleak formation.

### 3.3 TAWSS & OSI

**Figure 4:**
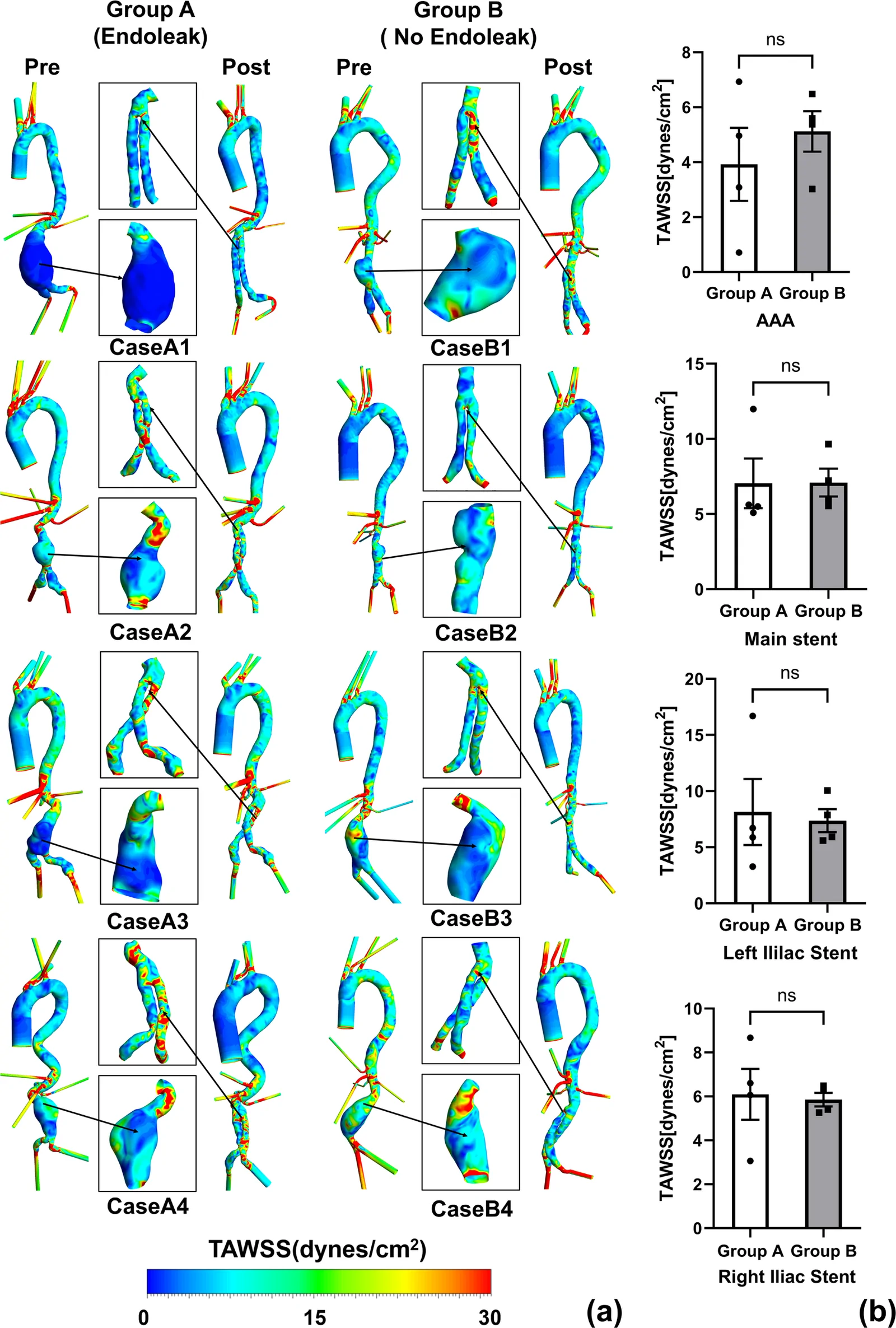
TAWSS: **(a)** TAWSS contours, with a general increase at the main stent and iliac branch stents postoperatively, with the stent junction regions identified as high-TAWSS regions, which was more pronounced in the endoleak group; **(b)** Comparison of the wall-averaged TAWSS, and the data was shown as mean ± SD.

TAWSS and OSI are critical hemodynamic parameters for assessing thrombosis risk and rupture potential in AAA. It is widely accepted that regions with low TAWSS are prone to thrombosis, whereas locations with high OSI and high TAWSS are associated with an increased risk of rupture. In this study, the preoperative AAA model exhibited a distinctly low TAWSS state, while the aneurysm neck showed relatively high TAWSS. Analysis of the wall-averaged TAWSS in the two patient groups revealed a general increase in TAWSS at the main stent and iliac branch stents postoperatively, with the stent junction regions identified as high-TAWSS regions. This phenomenon was more pronounced in the endoleak group—particularly in the left iliac artery stent, where the maximum TAWSS reached 16.7 dynes/cm². Notably, this observation aligned with the anatomical location of endoleaks, as illustrated in Figure 4a. However, comparison of the wall-averaged TAWSS at the main stent and iliac branch stents after EVAR between the two groups revealed no statistically significant difference. These findings suggest that TAWSS may lack sufficient sensitivity for the evaluation of Type I endoleaks.

**Figure 5:**
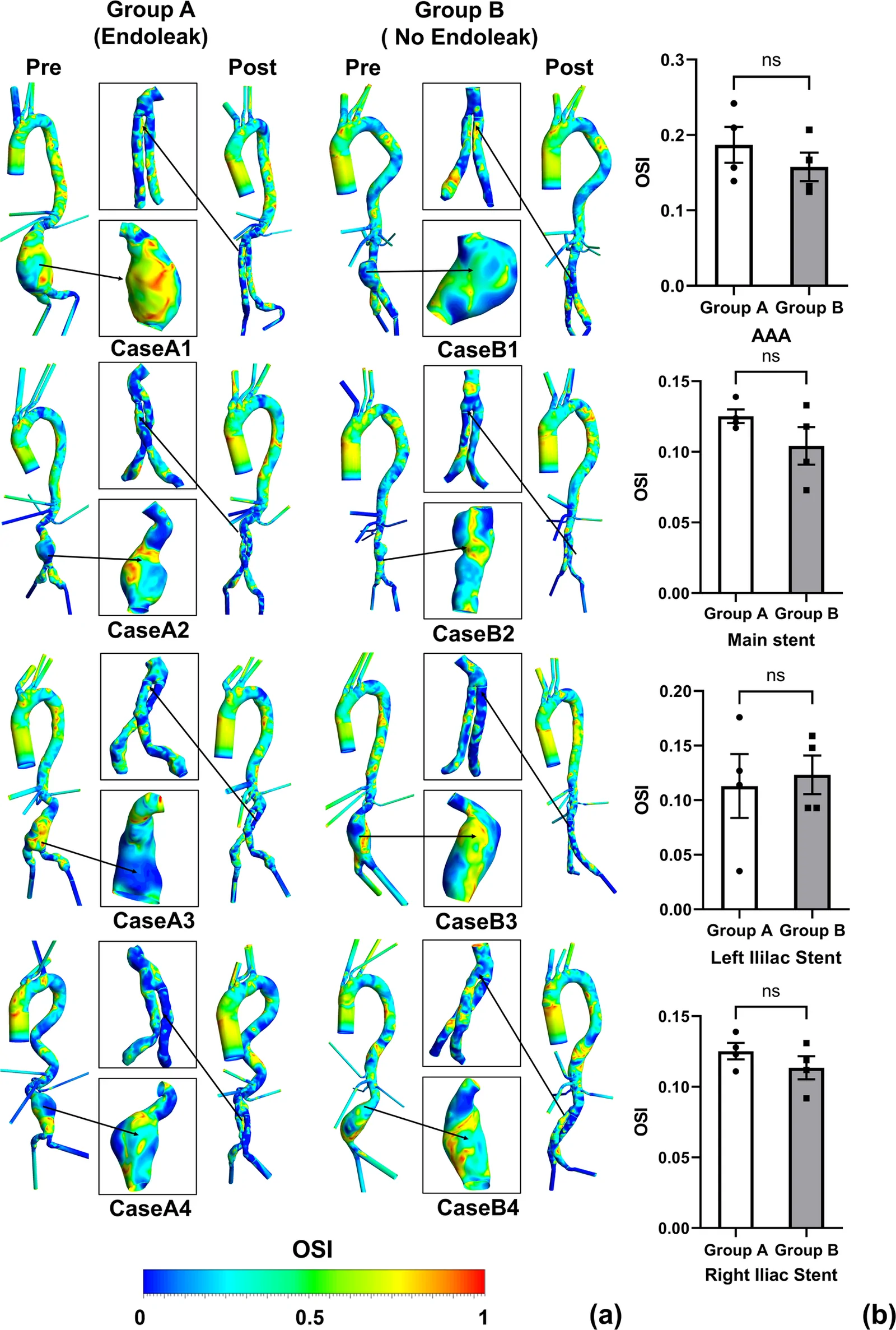
OSI**: (a)** OSI contours, with high-OSI regions persisted at the stent junctions, which corresponded to the locations of endoleaks; **(b)** Comparison of the wall-averaged OSI, and the data was shown as mean ± standard deviation (SD).

The distribution of OSI was shown in Figure 5a. In contrast to TAWSS, regions of the aneurysm wall with greater curvature typically exhibited higher OSI values. Following EVAR, OSI levels were generally reduced in the main stents and iliac branch stents; however, high-OSI regions persisted at the stent junctions, which corresponded to the locations of endoleaks. As shown in Figure 5b, although the wall-averaged OSI at the stent in the endoleak group was slightly higher than that in the non-endoleak group, no statistically significant difference was observed between the two groups. The maximum OSI value was only 0.176, measured at the left iliac branch stent of Case A1.

### 3.4 Energy loss

**Figure 6:**
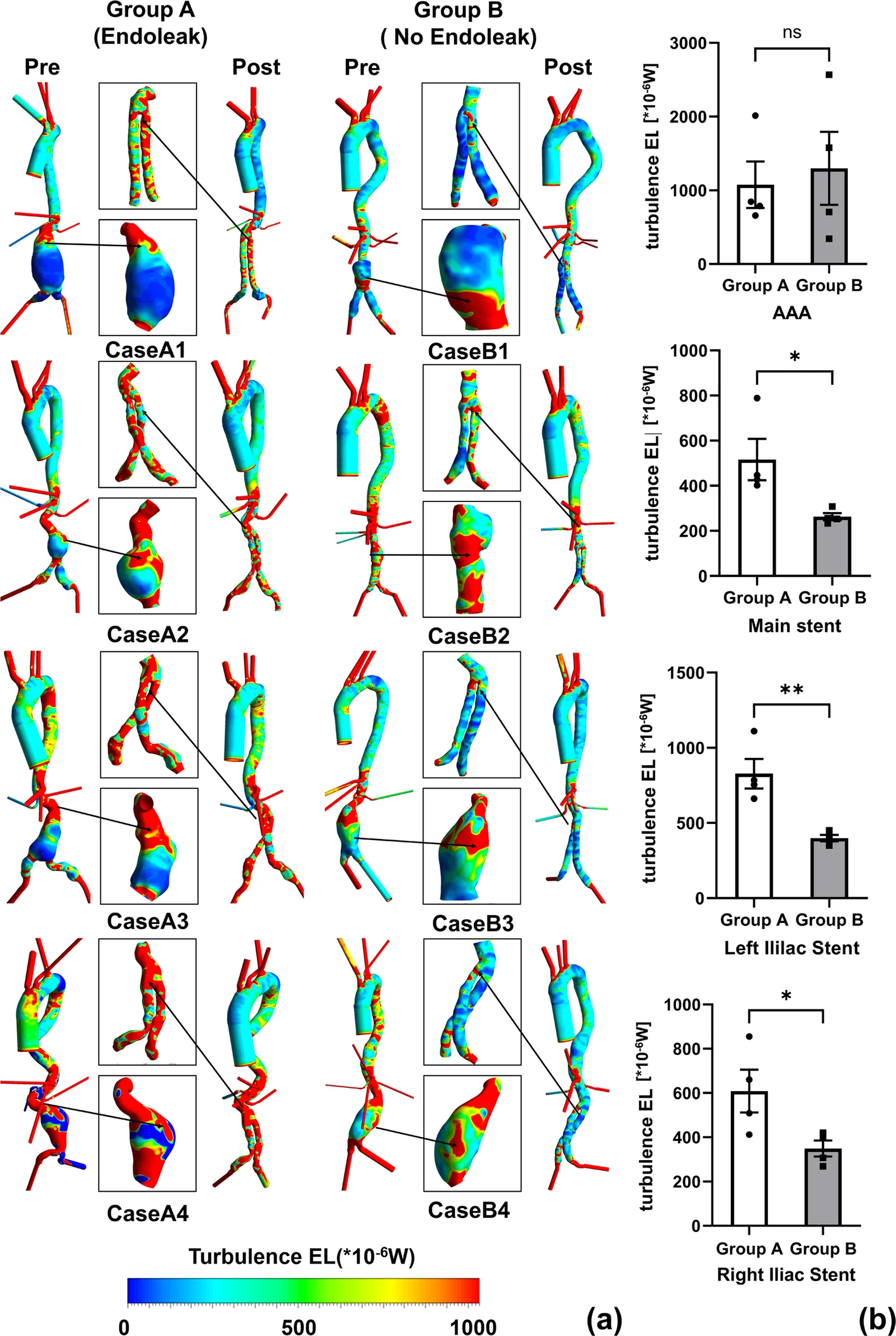
Turbulence EL: **(a)** turbulence EL contours, with the red parts of the main stents and the iliac branch stents in the endoleak group significantly more than those in the non-endoleak group; **(b)** the turbulence EL values at the main stents, left iliac artery stents, and right iliac artery stents in the endoleak group were all significantly higher than those in the non-endoleak group (p_main_ < 0.05, p_left_ < 0.01, p_righ_t < 0.05). (Data was shown as mean ± SD).

**Figure 7:**
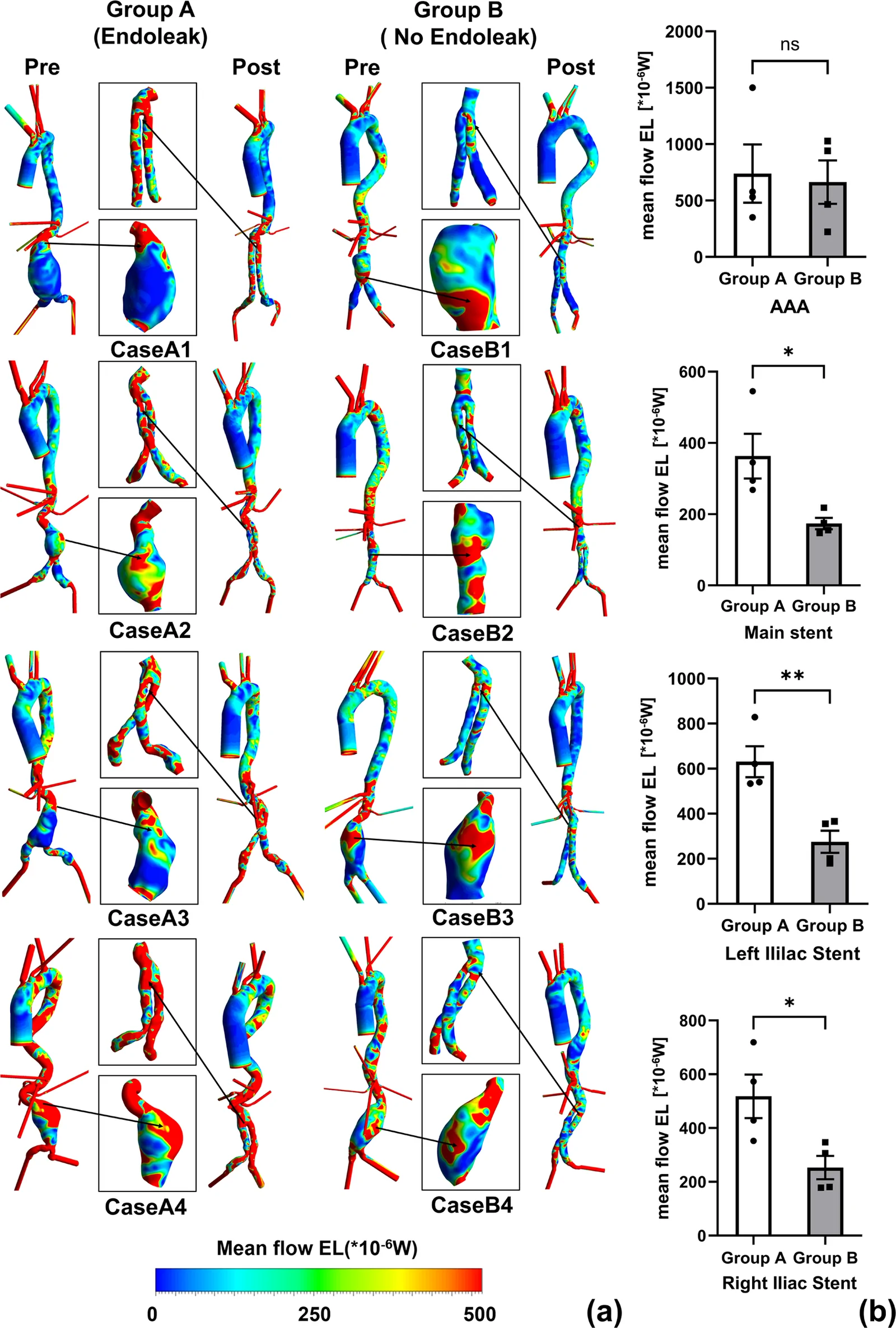
Mean flow EL: **(a)** mean flow EL contours, with the red parts of the main stents and the iliac branch stents in the endoleak group significantly more than those in the non-endoleak group; **(b)** the mean flow EL values at the main stents, left iliac artery stents, and right iliac artery stents in the endoleak group were all significantly higher than those in the non-endoleak group (p_main_ < 0.05, p_left_ < 0.01, p_righ_t < 0.05). (Data was shown as mean ± SD).

By using component quantification, the distribution of mean flow EL and turbulence EL was predicted and described in Figures 6a, 7a, respectively. In the preoperative model, regions of high energy loss were primarily localized at the aneurysm neck and segments with high curvature of the aneurysm. In the postoperative model, both mean flow EL and turbulence EL at the main stent and iliac artery stent were significantly higher in the endoleak group than in the non-endoleak group. In the non-endoleak group, high energy loss areas were only observed at the junction of the main stent and the iliac artery branch stents.

A comparative analysis of EL components revealed markedly elevated turbulence EL values in the endoleak group compared to the non-endoleak group across all stent positions (main: 516 ± 183 vs. 263 ± 32*10^−6^ W; left iliac: 828 ± 195 vs. 399 ± 43 *10^−6^ W; right iliac: 609 ± 193 vs. 349 ± 73 *10^−6^ W).The mean differences were 253 (95% CI: 25.7 to 480.3), 429 (95% CI: 184.7 to 673.3), and 260 (95% CI: 7.5 to 512.5) W·10^−6^, respectively, corresponding to very large effect sizes (Cohen's d = 1.93, 3.04, and 1.78).The turbulence EL values at the main stents, left iliac artery stents, and right iliac artery stents in the endoleak group were all significantly higher than those in the non-endoleak group (P_main_ < 0.05, P_left_ < 0.01, P_right_ < 0.05), as shown in Figure 6b.

Similarly, the mean flow EL values for the main stents, left iliac branch stents, and right iliac branch stents in the endoleak group were 363 ± 125, 631 ± 138, and 352 ± 162 *10^−6^ W, respectively, whereas those in the non-endoleak group were 174 ± 32, 276 ± 99, and 253 ± 87 *10^−6^ W. The mean differences between groups were 189 (95% CI: 31.1 to 346.9), 355 (95% CI: 147.2 to 562.8), and 99 (95% CI: −125.9 to 323.9) *10^−6^ W, corresponding to Cohen's d values of 2.07, 2.96, and 0.76, respectively. The mean flow EL values at the main stents, left iliac stents, and right iliac stents in the endoleak group were significantly higher than those in the non-endoleak group (P_main_ < 0.05, P_left_ < 0.01, P_right_ < 0.05). These findings suggested that mean flow EL and turbulence EL was associated with risk patterns and may support risk stratification the occurrence of Type I endoleak, as shown in Figure 7b.

## 4 Discussion

In this study, 8 patients with AAA who underwent EVAR were enrolled and stratified into two groups based on the presence or absence of Type I endoleak. Hemodynamic numerical simulations were performed on patient-specific pre- and post-operative models to calculate flow velocity, wall variables, and energy loss. Analysis of hemodynamic parameter distributions revealed that endoleaks predominantly occurred at stent junctions, where regions of high flow velocity, elevated TAWSS, increased OSI, and high energy loss were consistently observed. Comparative analysis of the two groups showed that while the flow velocity and wall-averaged TAWSS at the stents in the endoleak group were slightly higher than those in the non-endoleak group, no statistically significant differences were detected. However, component quantification of energy loss demonstrated that both turbulence EL and mean flow EL at the main stents and iliac branch stents were significantly higher in the endoleak group than in the non-endoleak group—particularly at the left iliac stents. This finding was consistent with the endoleak locations observed in four patients, indicating substantial energy loss in these regions (both on the stent surface and within the flow field) that was highly correlated with endoleak formation. In conclusion, a preliminary investigation involving 8 patients indicated that the quantitative assessment of energy loss components may hold feasibility for evaluating the formation of Type I endoleaks.

In prior studies, the occurrence of endoleaks has been primarily linked to morphological parameters and the mechanical properties of stents, yet the underlying mechanisms remain poorly investigated. In recent years, some researchers have employed WSS-related parameters to examine hemodynamic alterations on stent surfaces, revealing a correlation between endoleaks and elevated WSS (19–21), which was also validated in the present study. However, focusing solely on wall hemodynamic parameters may overlook the holistic dynamics of the flow field, leading to biased predictive outcomes. Consequently, the difference in TAWSS between the endoleak and non-endoleak groups was not statistically significant in this study. Separating energy loss into mean flow EL and turbulence EL for independent calculation facilitated the simultaneous assessment of flow field changes both on the stent surface and within its interior, enabling a more comprehensive and specific association with endoleak formation. Compared to conventional hemodynamic parameters, this approach offered enhanced accuracy and advantageous. Furthermore, in recent years, energy loss has been defined and applied to aortic diseases through alternative frameworks. Mo et al. (34) conceptualized energy loss as the irreversible conversion of mechanical energy to internal energy driven by friction between fluid micelles, and proposed a novel method to compute the equivalent electric field based on the dissipation function (DF) for predicting the rupture risk of intracranial aneurysms. Other studies have defined energy loss by collecting aneurysm tissues and performing *in vitro* tensile experiments, thereby establishing its correlation with rupture risk (35, 36). These investigations provide valuable insights for further exploring the relationship between energy loss and endoleak occurrence. Notably, CTA remains the gold standard for anatomical diagnosis of endoleak. The proposed EL component analysis is intended as a complementary tool for functional risk stratification rather than diagnostic replacement. Potential applications include identifying patients with adverse hemodynamic signatures who may benefit from closer surveillance or earlier intervention.

This study also has some limitations. First, a uniform time-varying waveform was employed at the inlet in the present study. This simplification may attenuate inter-individual hemodynamic variability and influence the absolute magnitude of EL, because EL is sensitive to local velocity gradients and flow disturbances. With the gradual popularization of ultrasonic velocity measurement and MRI 4D flow technology, patient-specific inlet flow rates could be acquired using these techniques in future research, thereby enhancing the realism of numerical simulations. Furthermore, numerical simulations need to consider the material properties of stent-grafts and arteries, including the rigidity and/or compliance of stent wall surfaces, the behavioral and/or physiological characteristics of intraluminal thrombus within the aneurysm, as well as patient-specific resistance at the distal end of the stent graft. These factors may affect pressure–flow coupling, local velocity gradients, and the storage, release, and dissipation of mechanical energy during the cardiac cycle, thereby potentially influencing EL estimation, and fluid–structure interaction (FSI) models may need to be implemented in future work to address these issues. Second, the inlet of the computational model was truncated in this study, which hindered the reproduction of the aortic sinus jet. In subsequent studies, the impact of the aortic sinus jet on hemodynamics should be taken into account when defining the inlet boundary conditions. Additionally, the simplification of models in this study may alter local pressure/velocity fields and potentially underestimate local recirculation/dissipation near endoleak-related regions, thus more effective methods need to be developed for predicting topology of endoleaks. Thirdly, although statistical significance of energy loss was observed between two groups, the extremely wide confidence intervals, attributable to the small sample size (*n* = 4 per group), warrant cautious interpretation of these findings. Finally, iliac artery branch stents in EVAR surgery are categorized into bifurcated stent-grafts and crossed-limbs based on their deployment methods. The differences in energy loss between these two approaches, as well as potential variations in endoleak formation, warrant further investigation. In addition, morphological variations, including aneurysm diameter, volume, shape, and neck angle, may also influence endoleak formation and energy dissipation. Future in-depth investigations should incorporate more cases and perform matched or paired group analyses based on relevant anatomical characteristics to enhance the scientific rigor and clinical relevance of the research.

## 5 Conclusion

In summary, the mean flow EL and turbulence EL at the main stents and iliac branch stents in the endoleak group were significantly higher than those in the non-endoleak group. Notably, regions of high energy loss were primarily concentrated at the stent junctions, which were consistent with the sites of endoleak occurrence. This suggests that the component quantification of energy loss holds the potential for evaluating Type I endoleak development, exhibiting greater accuracy and advantageous compared to conventional hemodynamic parameters. This method may offer valuable support to clinicians in assessing the efficacy and prognosis of EVAR in patients with AAA. However, due to geometric simplifications and limited sample size, these results should be considered hypothesis-generating and require prospective validation in larger cohorts.

## Data Availability

The original contributions presented in the study are included in the article/Supplementary Material, further inquiries can be directed to the corresponding authors.
